# Cost analysis of exercise cardiac magnetic resonance imaging in suspected dilated cardiomyopathy—a single-center experience

**DOI:** 10.1016/j.jocmr.2025.101924

**Published:** 2025-06-10

**Authors:** Sameera Senanayake, Sheryl Wei Xuan Lieo, Aisyah Binte Latib, Sanjeewa Kularatna, Nicholas Graves, Michelle Swee Leng Kui, Declan P. O’Regan, Mark Yan Yee Chan, Derek John Hausenloy, Calvin Woon Loong Chin, Thu-Thao Le

**Affiliations:** a Health Services and Systems Research Program, Duke-NUS Medical School, Singapore; b National Heart Research Institute Singapore, National Heart Centre Singapore, Singapore; c Clinical & Translational Research Office, National Heart Centre Singapore, Singapore; d Department of Cardiology, National Heart Centre Singapore, Singapore; e Medical Research Council Laboratory of Medical Sciences, Imperial College London, London, United Kingdom; f Department of Cardiology, National University Heart Centre, Singapore; g Yong Loo Lin School of Medicine, National University of Singapore, Singapore; h Cardiovascular Sciences Academic Clinical Program, Duke-NUS Medical School, Singapore; i Cardiovascular & Metabolic Disorders Program, Duke-NUS Medical School, Singapore; j The Hatter Cardiovascular Institute, University College London, London, United Kingdom

**Keywords:** Exercise cardiovascular magnetic resonance, Dilated cardiomyopathy, Cost effectiveness

## Abstract

**Background:**

Exercise cardiovascular magnetic resonance (ExCMR) imaging using supine in-scanner ergometer has shown promise in differentiating pathological dilated cardiomyopathy (DCM) from physiological exercise-induced cardiac remodeling. Since 2020, the National Heart Centre Singapore (NHCS) has incorporated ExCMR into its clinical workflow for patients with suspected DCM. This study aims to compare the costs associated with ExCMR versus conventional CMR in the evaluation of DCM.

**Methods:**

A retrospective analysis was conducted on patients referred for conventional CMR between 2016 and 2019, and those referred for ExCMR from 2020 to 2023. Both imaging modalities followed standardized protocols, with ExCMR incorporating additional assessments during peak exercise. Costs were recorded in Singapore dollars (SGD) prior to the application of healthcare subsidies.

**Results:**

The total cost for conventional CMR was SGD 1831.36, while ExCMR was associated with a higher initial cost of SGD 2336.48. However, ExCMR resulted in significantly fewer abnormal imaging findings and a reduced need for follow-up investigations (6.5% (9/139) vs 56.8% (71/125), p<0.001). A decision tree analysis and probabilistic sensitivity analysis (PSA) revealed that diagnosing 1000 suspected DCM patients with ExCMR could result in a cost savings of approximately SGD 182,323 compared to conventional CMR, with a 64% probability of being cost-effective.

**Conclusion:**

These findings indicate that ExCMR offers a physiologically informative approach for diagnosing DCM, with the potential to reduce overdiagnosis of cardiac dilatation in active, healthy adults. Although further research is necessary to assess long-term outcomes, ExCMR appears to be a cost-effective imaging modality for DCM diagnosis, warranting reconsideration of its perceived higher cost.

## Graphical abstract

**Figure:**
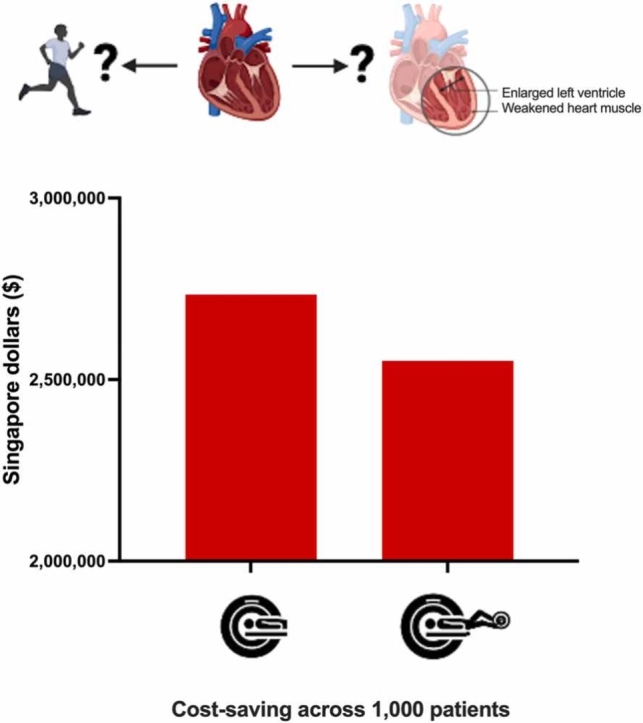

## 1. Introduction

Exercise cardiac magnetic resonance (ExCMR) imaging using supine in-scanner ergometer has shown promise in distinguishing between pathological dilated cardiomyopathy (DCM) and physiological exercise-induced cardiac remodeling [1], [2]. The National Heart Centre Singapore (NHCS) has integrated this novel diagnostic approach into its clinical workflow since 2020 for patients with suspected DCM.

In clinical practice, initial assessment often includes echocardiogram (echo). While it is a potentially useful and affordable tool, it is highly operator-dependent and lacks the ability to characterize myocardial tissue—an important feature for differentiating DCM from physiological remodeling [3]. In contrast, CMR provides unique markers such as the presence of late gadolinium enhancement (LGE), elevated native T1 values, and increased extracellular volume fraction (ECV), which indicate myocardial fibrosis typical of pathological DCM, but are generally absent in physiological adaptation [4].

ExCMR enables simultaneous evaluation of both myocardial function under stress and tissue composition. Despite its clinical potential, data on the cost efficiency of ExCMR remain scarce, with only a single published study evaluating cost implications using treadmill ExCMR for ischemic assessment [5]. Given the increasing need for cost-effective diagnostic strategies, a more comprehensive assessment of ExCMR’s financial impact is warranted. From this single-center experience, we compared the costs associated with ExCMR versus conventional CMR for the evaluation of DCM.

## 2. Method

### 2.1. Study population

Individuals who were referred had a clinical history of suspected DCM with clinical symptoms, abnormal echo (dilated ventricle and/or impaired ventricular function), and/or abnormal electrocardiogram (ECG) results [3], [6]. Exclusion criteria included ischemic heart disease, hypertension, and valvular disease. The analysis included individuals referred for conventional CMR without undergoing subsequent ExCMR from 2016 to 2019, as well as those referred directly for ExCMR from 2020 to 2023. The study was approved by the SingHealth Centralized Institutional Review Board and conducted in accordance with the Declaration of Helsinki. Written informed consent was obtained from all participants undergoing ExCMR, while informed consent was waived for those undergoing conventional CMR.

### 2.2. Cardiovascular magnetic resonance imaging

All participants underwent standardized resting cine and LGE imaging (Siemens Aera 1.5T, Erlangen, Germany). ExCMR was performed with an in-scanner supine ergometer (Lode BV, Groningen, Netherlands) using a published protocol [1], [7]. In brief, the protocol includes conventional CMR imaging acquisition at rest and every stage of exercise until exhaustion. Cardiac volumes at rest (breath-hold short-axis cine images) and each exercise stage (free-breathing real-time short-axis cine images) were assessed using CVI42 auto-segmentation algorithm with visual inspection and manual adjustment as necessary (version 5.14, Circle Cardiovascular Imaging Inc., Calgary, Alberta, Canada). Rest and peak cardiac indices (CI) were calculated and expressed as age- and sex-specific percentiles [1].

Referring physicians received standardized reports with detailed imaging findings and exercise. In the conventional CMR group, abnormal imaging finding was defined by any of the following criteria: (i) cardiac dilatation, (ii) impaired ventricular function based on age- and sex-specific Asian CMR reference values [8], and (iii) presence of LGE enhancement. In the ExCMR group, an abnormal result was determined by at least one abnormal imaging findings at rest—using the same criteria as the conventional CMR group—and impaired exercise capacity, indicated by peak cardiac index below 35th percentile of age- and sex-specific CMR-derived reference range from our center, following adequate exercise (with a normal heart rate response) [1]. Patients with low exercise capacity but normal imaging findings at rest were classified as normal with a low fitness level.

### 2.3. Cost effectiveness and statistical analyses

In each patient, the costs of CMR scan, follow-up outpatient clinic visits, and follow-up investigations, including ECG, echo, cardiopulmonary test (CPET), and repeat CMR prior to discharge or referral to cardiomyopathy clinic, were recorded up to November 2024. Follow-up decisions were made by primary physicians based on a combination of medical history and CMR report. The costs were reported in Singapore dollars (SGD), before healthcare subsidies and financing schemes. The total costs of conventional CMR and ExCMR were $1831.36 and $2336.48, respectively.

**Fig. 1:**
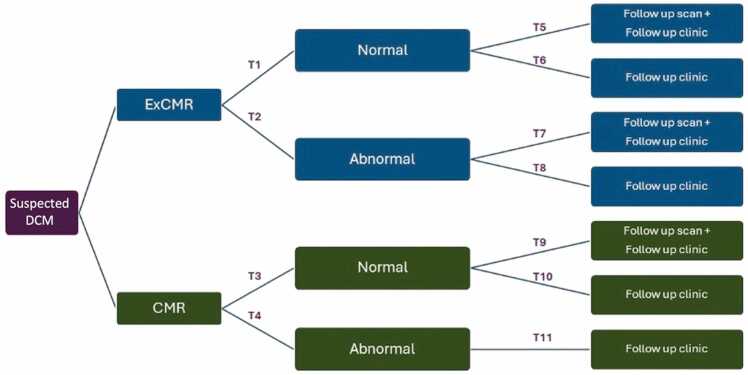
Decision tree outlining the sequence of clinical decisions prior to discharge or referral to cardiomyopathy clinic. *ExCMR* exercise cardiovascular magnetic resonance, *DCM* dilated cardiomyopathy, *CMR* cardiovascular magnetic resonance.

Depending on the normality of the data, differences in continuous variables were compared using either parametric Student’s t test or the nonparametric Mann–Whitney U test. Categorical variables were compared using the χ^2^ test. A two-sided p value <0.05 was considered statistically significant. The decision tree was created to outline the sequence of clinical decisions and the associated costs of ExCMR and conventional CMR (Fig. 1). Costs and transition probabilities were estimated for each group. Probabilistic sensitivity analysis (PSA) was used to assess the uncertainty in the parameters used in the model, and their effect on the cost-analysis results. Monte Carlo simulations were performed with 10000 iterations. The incremental costs associated with both diagnostic pathways were simulated using 1000 patients transitioning through the decision tree. The PSA parameters were input into the model to account for potential uncertainty. Statistical and cost analyses were performed using SPSS Statistics, version 28 (IBM, Armonk, New York) and TreeAge Pro (Williamstown, Massachusetts).

## 3. Results

The study population consisted of 139 subjects (24 ± 10 years, 98.6% males) who underwent ExCMR and 125 subjects (21 ± 5 years, 99.2% males) who underwent conventional CMR (Table 1). Of all patients, 165 (62.5%) underwent rest and/or stress echo, or CPET assessment prior to referral for CMR. Compared to conventional CMR, the probability of abnormal imaging findings after ExCMR was significantly lower (0.134 ± 0.031 vs 0.789 ± 0.042, p<0.001) and fewer subjects required additional follow-up investigations (6.5% (9/139) vs 56.8% (71/125), p<0.001).

**Table 1:** Clinical characteristics, CMR findings, and follow-up visits of patients underwent conventional versus exercise CMR.

	Conventional CMR (n = 125)	ExCMR (n = 139)	P Value
Clinical Characteristics			
Age (years)	21 ± 5	25 ± 10	<0.001
Males, n (%)	124 (99.2)	137 (98.6)	0.626
Body mass index (kg/m2)	24.2 ± 6.3	23.9 ± 4.5	0.691
Heart rate (bpm)	71 ± 19	65 ± 14	0.010
Systolic blood pressure (mmHg)	133 ± 16	115 ± 12	<0.001
Diastolic blood pressure (mmHg)	75 ± 13	65 ± 12	<0.001
CMR Findings			
Dilated LV, n (%)	51 (40.8)	43 (30.9)	0.095
Impaired LV ejection fraction, n (%)	35 (28.0)	29 (20.9)	0.177
Presence of LGE, n (%)	15 (12.0)	14 (10.1)	0.618
Follow-up after CMR			
Referral to cardiomyopathy clinic, n (%)	33 (26.4)	19 (13.7)	0.009
Additional follow-up visits, n (%)	71 (56.8)	9 (6.5)	<0.001
Non-stress imaging test, n (%)	38 (30.4)	1 (0.7)	<0.001
Stress test, n (%)	34 (27.2)	2 (1.4)	<0.001

*ExCMR* exercise cardiovascular magnetic resonance, *LV* left ventricular

The average cost for diagnosing 1000 suspected DCM patients with ExCMR was $2,551,795. In contrast, the conventional CMR cost for the same number of patients was $2,734,118, indicating a cost-saving of $182,323 for ExCMR. PSA indicates that the probability of ExCMR being cost-saving is 65%.

## 4. Discussion

Exercise-induced cardiac remodeling extends beyond athletes and can occur in active, healthy adults, raising concerns about the potential for over-diagnosing cardiac dilatation in this population [9]. While specific reference ranges for athletes have been proposed to differentiate physiological adaptation from cardiomyopathy, these recommendations remain limited by data scarcity and high variability due to factors such as race, age, and training intensity [10]. As a result, distinguishing benign exercise-induced changes from early-stage DCM remains a diagnostic challenge, particularly in physically active individuals undergoing cardiovascular screening. This study provides important clinical and economic insights by demonstrating that ExCMR has the potential to reduce unnecessary diagnoses of DCM while offering a more cost-effective alternative to conventional rest CMR. The ability to assess cardiac function under physiological stress conditions enhances diagnostic accuracy, which may reduce the need for repetitive imaging, follow-up investigations, and unnecessary referrals, ultimately lowering the overall healthcare burden.

While the long-term effectiveness of the ExCMR approach requires further evaluation, our findings strongly support for its role as a cost-effective and physiologically informative imaging modality for diagnosing DCM, particularly when initial tests like echocardiography and CPET are inconclusive. Approximately 60% of our cohort underwent rest/stress echo or CPET before CMR referral, highlighting their diagnostic limitations. While more affordable, neither test can detect LGE, a key feature in differentiating pathological from physiological remodeling[3]. By enabling simultaneous assessment of myocardial function under stress and tissue characterization, ExCMR enhances diagnostic confidence. At our center, this integration resulted in a follow-up testing rate of only 6.5%, suggesting efficient resource use.

Additionally, the perception of ExCMR as costly should be re-evaluated in light of these results, which endorse ExCMR as a lower-cost modality. Integrating ExCMR into routine diagnostic pathways may optimize resource utilization and improve the early identification of true pathological cases of DCM, preventing both overdiagnosis and overtreatment.

## 5. Conclusion

ExCMR offers a promising, cost-effective alternative to conventional CMR by reducing the risk of over-diagnosing exercise-induced cardiac remodeling in active adults. Further research is needed to validate its long-term clinical utility, but these findings support its broader adoption for diagnosing DCM.

## Funding

This study was supported by the Singapore National Medical Research Council (grant MOH-000956–00) and the CVD Health Services Research (HSR) unit under the CArdiovascular DiseasE National Collaborative Enterprise (CADENCE) National Clinical Translational Program (MOH-001277).

## Author contributions

**Sameera Senanayake:** Writing – original draft, Formal analysis, Data curation. **Sheryl Wei Xuan Lieo:** Writing – review & editing, Visualization, Data curation. **Aisyah Binte Latib:** Writing – review & editing, Data curation. **Sanjeewa Kularatna:** Writing – review & editing, Methodology. **Nicholas Graves:** Writing – review & editing, Supervision. **Michelle Swee:** Leng Kui Writing – review & editing. **Declan P. O’Regan:** Writing – review & editing. **Mark Yan Yee Chan:** Writing – review & editing, Funding acquisition. **Derek John Hausenloy:** Writing – review & editing, Funding acquisition. **Calvin Woon Loong Chin:** Writing – review & editing, Supervision, Methodology, Conceptualization. **Thu-Thao Le:** Writing – original draft, Funding acquisition, Formal analysis, Data curation, Conceptualization.

## Declaration of competing interests

The authors declare that they have no known competing financial interests or personal relationships that could have appeared to influence the work reported in this paper.
